# Thyroid dysfunction in metabolic syndrome patients and its relationship with components of metabolic syndrome

**DOI:** 10.1186/s40842-016-0021-0

**Published:** 2016-02-01

**Authors:** Saroj Khatiwada, Santosh Kumar Sah, Rajendra KC, Nirmal Baral, Madhab Lamsal

**Affiliations:** 1 Department of Pharmacy, Central Institute of Science and Technology (CIST) College, Pokhara University, Kathmandu, Nepal; 2 Department of Biochemistry, Universal College of Medical Sciences, Bhairahawa, Nepal; 3 Department of Medical Laboratory Technology, Modern Technical College, Satdobato, Lalitpur, Nepal; 4 Department of Biochemistry, B P Koirala Institute of Health Sciences, Dharan, Nepal

**Keywords:** Metabolic syndrome, Nepal, Subclinical hypothyroidism, Thyroid dysfunction

## Abstract

**Background:**

A growing body of evidence suggests that metabolic syndrome is associated with endocrine disorders including thyroid dysfunction. Thyroid dysfunction in metabolic syndrome patients may further add to cardiovascular disease risk thereby increasing mortality. This study was done to assess thyroid function in metabolic syndrome patients and evaluate its relationship with the components of metabolic syndrome.

**Methods:**

A cross sectional study was carried out among 169 metabolic syndrome patients at B P Koirala Institute of Health Sciences, Dharan, Nepal. Anthropometric measurements (height, weight, waist circumference) and blood pressure were taken. Fasting blood samples were analysed to measure glucose, triglyceride, high density lipoprotein (HDL) cholesterol and thyroid hormones (triiodothyronine, thyroxine and thyroid stimulating hormone).

**Results:**

Thyroid dysfunction was seen in 31.9 % (*n* = 54) metabolic syndrome patients. Subclinical hypothyroidism (26.6 %) was the major thyroid dysfunction followed by overt hypothyroidism (3.5 %) and subclinical hyperthyroidism (1.7 %). Thyroid dysfunction was much common in females (39.7 %, *n* = 29) than males (26 %, *n* = 25) but not statistically significant (*p* = 0.068). The relative risk of having thyroid dysfunction in females was 1.525 (CI: 0.983–2.368) as compared to males. Significant differences (*p* = 0.001) were observed in waist circumference between patients with and without thyroid dysfunction and HDL cholesterol which had significant negative correlation with thyroid stimulating hormone.

**Conclusions:**

Thyroid dysfunction, particularly subclinical hypothyroidism is common among metabolic syndrome patients, and is associated with some components of metabolic syndrome (waist circumference and HDL cholesterol).

## Background

Metabolic syndrome constitutes a cluster of risk factors characterized by hypertension, atherogenic dyslipidemia, hyperglycemia, prothrombotic and proinflammatory conditions [1]. This cluster of metabolic abnormalities is associated with increased risk for atherosclerotic cardiovascular disease and type 2 diabetes mellitus [2]. The prevalence of metabolic syndrome is increasing all over the world with distinct evidence of high prevalence in India and other South Asian countries [3].

Thyroid dysfunction, prominently subclinical hypothyroidism has been observed more frequently in metabolic syndrome patients than general population [3]. Both metabolic syndrome and hypothyroidism are independent risk factors for cardiovascular diseases (CVD). Presence of both conditions may be compounded to increase the risk for CVD and a considerable overlap occurs in the pathogenic mechanisms of atherosclerotic cardiovascular disease by metabolic syndrome and hypothyroidism [4]. There are reports about higher thyroid stimulating hormone (TSH) level in metabolic syndrome patients than in healthy ones, and high prevalence of metabolic syndrome in subjects with TSH level higher than normal as compared to those with normal TSH level [5, 6]. However the association between thyroid dysfunction and components of metabolic syndrome is still debatable [7].

There is evidence that thyroid function may need to be assessed in patients with metabolic syndrome who are also at higher risk for CVD. Thyroid dysfunction is common in Nepal, and the prevalence of diabetes mellitus and metabolic syndrome has been rising steadily. Reports suggest that 20.7 % of the Nepalese population have metabolic syndrome based on National Cholesterol Education Program (NCEP) criteria [8, 9]. However, thyroid function in such patients is not well studied. A study by Gyawali et al. in the central region of Nepal reported thyroid dysfunction in 31.8 % of metabolic syndrome patients [3]. We conducted the present study among metabolic syndrome patients in the eastern region of Nepal to assess the rate of thyroid dysfunction and explore the potential relationship between components of metabolic syndrome and thyroid function, and provide evidence for the better clinical management of metabolic syndrome patients. The present study aims to evaluate the pattern of thyroid dysfunction in Nepalese metabolic syndrome patients.

In this study, we assessed thyroid function and examined the association between components of metabolic syndrome and thyroid function in Nepalese population with metabolic syndrome.

## Methods

A cross-sectional study was conducted among metabolic syndrome patients at the department of biochemistry of B P Koirala Institute of Health Sciences (BPKIHS), Dharan, Nepal from September 2013 to September 2014. One hundred sixty nine metabolic syndrome patients aged ≥20 years were selected from the hospital during the study period. The sample size was calculated by taking prevalence of thyroid dysfunction as 15 % (approximate) in this region. Metabolic syndrome was diagnosed based on modified Asian NCEP-ATP III panel criteria [10]. The exclusion criteria was patients receiving medication that may alter thyroid functions or lipid levels, pregnant women, and patients with a cardiovascular disease, corticosteroid use, active liver disease, and renal dysfunction. After taking informed consent from each patient, height, weight, waist circumference and blood pressure was taken from each subject. The study protocol was approved by the institute review board of B P Koirala Institute of Health Sciences. Fasting venous blood samples (5 ml) were collected and analyzed for blood glucose, triglycerides, high density lipoprotein (HDL) cholesterol, free triiodothyronine (T3), free thyroxine (T4) and thyroid stimulating hormone (TSH). Blood glucose and triglycerides were measured by enzymatic method (kits from AGAPPE diagnostics by Biolyzer 100) and HDL cholesterol by homogeneous, direct method (kits from Gesan by Biolyzer 100). Serum free T3, free T4 and TSH were measured by using fluorescent immunoassay (VIDAS, biomeriux SA, France).

Metabolic syndrome patients were considered to have thyroid dysfunction if patients thyroid hormones level fell outside the reference range (free T3 (4.0–8.3 pmol/L), free T4 (9.0–20.0 pmol/L) and TSH level (0.25–5 mIU/L)). Patients were said to be euthyroid if all thyroid hormone levels fell within reference range. Overt hypothyroidism was defined as TSH > 5 mIU/L and free T3 < 4.0 pmol/L and free T4 < 9.0 pmol/L. Subclinical hypothyroidism was considered if TSH > 5 mIU/L and free T3 and free T4 within reference range. Subclinical hyperthyroidism was defined as TSH < 0.25 mIU/L and free T3 and free T4 within reference range. The data generated from study was analyzed using SPSS version 11.0. Continuous variables were expressed as mean ± SD values. Independent sample t test and one way ANOVA was applied for continuous variables and chi square test for categorical variables at 95 % confidence interval. Pearson correlation coefficients were calculated to find relationship between the components of metabolic syndrome and thyroid profile parameters at 95 % confidence interval. Relative risk with 95 % confidence interval (CI) was used to assess the risk factors for thyroid dysfunction in metabolic syndrome.

## Results

The study population consisted of 56.8 % (*n* = 96) males and 43.2 % (*n* = 73) females, with mean age of 47 ± 12.5 years. Height, weight, body mass index (BMI), waist circumference, systolic BP and diastolic BP were 157.4 ± 8 cm, 70.7 ± 7.9 Kg, 28.6 ± 3.3 Kg/m^2^, 102.5 ± 6.7 cm, 129.3 ± 13.6 mmHg and 84.9 ± 11.5 mmHg respectively. Similarly, levels of biochemical parameters; fasting blood glucose, triglyceride, HDL cholesterol, free T3, free T4 and TSH were 126.2 ± 50.4 mg/dL, 198.2 ± 90.8 mg/dL, 49.9 ± 15.3 mg/dL, 5.1 ± 1.0 pmol/L, 12.0 ± 2.9 pmol/L and 4.2 ± 3.4 mIU/L respectively. Thyroid dysfunction was found in 31.9 % (*n* = 54) patients. Subclinical hypothyroidism (26.6 %) was the major thyroid dysfunction followed by overt hypothyroidism (3.5 %) and subclinical hyperthyroidism (1.7 %). Components of metabolic syndrome according to thyroid dysfunction type are shown in Table 1. Waist circumference and blood glucose was significantly different across the thyroid dysfunction groups. Thyroid dysfunction was more common among females 39.7 % (*n* = 29) than males 26 % (*n* = 25) but not statistically significant (*p* = 0.068). Among males, 23 had subclinical hypothyroidism, 1 had overt hypothyroidism and 1 had subclinical hyperthyroidism. Similarly among females, 22 had subclinical hypothyroidism, 5 had overt hypothyroidism and 2 had subclinical hyperthyroidism. The relative risk of having thyroid dysfunction in females was 1.525 (0.983–2.368; *p* = 0.068) as compared to males. When metabolic syndrome parameters were compared between the patients subgroups (with TSH < 5 mIU/L and TSH ≥ 5 mIU/L), then systolic BP, diastolic BP, waist circumference, blood glucose, triglyceride and HDL cholesterol were 129.7 ± 12.6 mmHg versus 128.4 ± 16 mmHg; *p* = 0.602, 85.3 ± 11 mmHg versus 84 ± 12.8 mmHg; *p* = 0.513, 103.4 ± 6.6 cm versus 100.5 ± 6.6 cm; *p* = 0.008, 125.2 ± 53.2 mg/dL versus 128.6 ± 43.7 mg/dL; *p* = 0.692, 189.7 ± 75.5 mg/dL versus 217.9 ± 117.6 mg/dL; *p* = 0.119 and 51 ± 16.8 mg/dL versus 47.4 ± 11 mg/dL; *p* = 0.103 respectively. Correlation between the components of metabolic syndrome and TSH and free T4 is shown in Table 2. HDL cholesterol showed significant negative correlation with TSH level. BMI had negative correlation with free T3 (r = -0.161, *p* = 0.037) and free T4 (r = -0.15, *p* = 0.052) and weak positive correlation with TSH (r = 0.105, *p* = 0.174).

**Table 1:** Components of metabolic syndrome among different thyroid dysfunction group

Parameters	Euthyroid *N* = 115	Overt hypothyroid *N* = 6	Subclinical hypothyroid *N* = 45	Subclinical hyperthyroid *N* = 3
Systolic BP (mmHg)	129.7 ± 12.7	130 ± 10.9	128.2 ± 16.6	131.6 ± 2.8
Diastolic BP (mmHg)	85.5 ± 10.9	85.8 ± 15.3	83.7 ± 12.5	76.7 ± 15.2
Waist circumference (Cm)	103.7 ± 6.4	99.3 ± 8.5	100.6 ± 6.4	92.3 ± 4.6
Blood glucose (mg/dL)	123 ± 51	106.1 ± 32.2	131.5 ± 44.4	209.6 ± 75.1
Triglyceride (mg/dL)	190.5 ± 75.7	201.6 ± 69	220.1 ± 123	158 ± 75.7
HDL cholesterol (mg/dL)	51 ± 17	50 ± 12.4	47 ± 10.9	49.3 ± 1.1

**Table 2:** Correlation of components of metabolic syndrome with TSH and free T4

Components	TSH		Free T4	
	R	*P* value	R	*P* value
Systolic BP (mmHg)	-0.001	0.985	0.062	0.421
Diastolic BP (mmHg)	0.077	0.321	-0.141	0.068
Waist circumference (Cm)	-0.121	0.117	-0.056	0.469
Blood glucose (mg/dL)	0.059	0.444	-0.051	0.513
Triglyceride (mg/dL)	0.064	0.408	-0.035	0.65
HDL cholesterol (mg/dL)	-0.174	0.024	0.1	0.195

## Discussion

Metabolic syndrome can be associated with endocrine and non-endocrine disorders and has widespread consequences. Alterations in thyroid functions, though well known, are not recognized clinically and there is inconsistency in thyroid functions in metabolic syndrome [11]. The present study identifies thyroid dysfunction as a common endocrine disorder in metabolic syndrome patients; subclinical hypothyroidism (26.6 %) was the commonest followed by overt hypothyroidism (3.5 %) and subclinical hyperthyroidism (1.7 %). Our findings are consistent with previous studies investigating thyroid function in metabolic syndrome patients. A study by Gyawali et al. in Kavre district of central Nepal reported thyroid dysfunction in 31.84 % of metabolic syndrome patients, the most common dysfunction was subclinical hypothyroidism (29.32 %) followed by overt hypothyroidism (1.67 %) and subclinical hyperthyroidism (0.83 %) [3]. Previous studies in eastern Nepal have also reported higher rate of thyroid dysfunction. Though, thyroid function status of adult population from cross-sectional studies in community settings are unavailable, hospital based studies by Baral et al. and Khatiwada et al. have reported higher rate of thyroid disorders [8, 12]. Study by Baral et al. in eastern Nepal reported hyperthyroid and hypothyroid in 13.68 % and 17.19 % of the general population respectively. Similarly, Khatiwada et al. observed thyroid dysfunction in 36 % of diabetes mellitus patients in a tertiary hospital of eastern Nepal. Prevalence of higher rates of thyroid dysfunction in this region may be due to higher rate of thyroid autoimmunity, iodine deficiency or iodine excess [12]. Recent findings about iodine nutrition among children of this region indicate excess iodine intake in these areas as revealed by excess urinary iodine excretion [13]. A study in India by Shantha et al. found subclinical hypothyroidism in 21.9 % and overt hypothyroidism in 7.4 % metabolic syndrome patients [1]. Similarly, a study by Meher et al. showed a high prevalence of subclinical hypothyroidism (22 %) and overt hypothyroidism (4 %) in the metabolic syndrome patients [14]. We observed thyroid dysfunction to be more common in females (39.7 %) than males (26 %) patients, and this has been observed in a number of studies including the general population [1].

The TSH level of metabolic syndrome patients in our study was in upper normal range, which suggests some degree of thyroid dysfunction in such patients. In a case control study assessing CVD risk factors in an eastern Nepalese population, the mean TSH level of a healthy control population was 2.35 ± 1.07 mIU/L, which is lower than the mean TSH of this present study [15]. The TSH level was above the reference range for normal population in the study of Gyawali et al., and they also observed significantly higher TSH level in metabolic syndrome patients as compared to controls [3]. A positive association has also been reported, between a higher TSH level within the euthyroid reference range and the prevalence of the metabolic syndrome [5]. A study in Korea indicated that higher levels of TSH may predict the metabolic syndrome in the study subjects, suggesting that the influence of thyroid function on metabolic abnormality extends into subjects without metabolic syndrome [16]. In the present study, subclinical hyperthyroid patients had highest systolic BP and fasting blood glucose, overt hypothyroid patients had highest diastolic BP and euthyroid patients had highest waist circumference. Highest triglyceride and lowest HDL cholesterol was seen in patients with subclinical hypothyroidism. It has been observed that subclinically hypothyroid patients have higher systolic BP, diastolic BP, fasting blood glucose and triglycerides compared to euthyroid patients [17]. In our previous case control study, we observed that subclinical hypothyroidism patients have significantly higher diastolic BP, total cholesterol, LDL cholesterol and hs-CRP than euthyroid controls [15]. Our current findings may be due to small number of thyroid dysfunction patients (overt hypothyroidism and subclinical hyperthyroidism). Hypothyroidism is associated with factors of metabolic syndrome such as dyslipidemia, hypertension, obesity, and often insulin resistance. It has been reported that 95 % of newly diagnosed hypothyroid patients have increased levels of cholesterol and 5 % of have hypertriglyceridemia. Hypothyroidism also leads to increased level of LDL cholesterol. All these factors directly contribute to accelerated atherosclerosis [18].

The correlation between subclinical hypothyroidism and metabolic syndrome and its components varies in different studies and seems to be influenced by age, gender and race of study participants [19]. In the present study, waist circumference was significantly different (*p* = 0.001) between patients with and without thyroid dysfunction, and HDL cholesterol had significant negative association with TSH level, however, other components of metabolic syndrome had no significant relationships with thyroid dysfunction. Thyroid hormones affect lipid metabolism and thus the components of metabolic syndrome, and there is positive relation between TSH and LDL cholesterol, whereas negative relation between TSH and HDL cholesterol [15]. Our findings are similar to previous study, where no significant relationship between components of metabolic syndrome and thyroid dysfunction were found except for waist circumference [3]. There are contrasting reports about the association between various metabolic syndrome parameters and thyroid function. In a study in Nigeria, metabolic syndrome was significantly associated with higher free T4 levels [2]. In a study in India, subclinical hypothyroidism was significantly associated with metabolic syndrome and a linear association was observed between TSH levels and total cholesterol, triglycerides, LDL, and HDL cholesterol levels across the metabolic syndrome group [14]. However, in a study in Turkey, TSH was not related with any metabolic syndrome parameters [20].

In the present study, we observed negative association of BMI with free T3 and free T4 and weak positive correlation with TSH. In a study in Germany, euthyroid subjects with TSH in the upper normal range (2.5–4.5 mU/L) were more obese (BMI > 30 Kg/m^2^), had higher triglyceride levels, and an increased likelihood of having metabolic syndrome [21].

High prevalence of overt and subclinical hypothyroidism in metabolic syndrome as seen in our study may have harmful effect on cardiovascular health. Hypothyroidism will lead to increased lipid levels and hypertension leading to increased risk for CVD. The effects due to metabolic syndrome and hypothyroidism may be compounded to increase risk for CVD [1]. Thus, assessing thyroid function in metabolic syndrome patients may help identify patients at high risk for CVD. However, it is still unclear whether patients with subclinical hypothyroidism should be treated, and the distinct benefit of prescribing levothyroxine for cardiovascular benefits in these patients is still debatable [15]. The present study has however several limitations. First the sample size was small, which may have affected the correlation between components of metabolic syndrome and thyroid function. Second, the iodine nutrition status in the patients was not assessed. It has been found that both iodine deficiency and excess can lead to thyroid disorder particularly subclinical hypothyroidism. Also, the presence of thyroid autoimmunity in the study population may lead to higher rate of thyroid dysfunction [12].

## Conclusions

In conclusion, the study finds thyroid dysfunction specifically subclinical hypothyroidism is a common endocrine disorder in Nepalese patients with metabolic syndrome, and thyroid function is associated with certain components of metabolic syndrome (waist circumference and HDL cholesterol).

## Abbreviations

BMI: Body mass index
BP: Blood pressure
CVD: Cardiovascular disease
Free T3: Free triiodothyronine
Free T4: Free thyroxine
hs-CRP: High sensitivity C reactive protein
HDL: High density lipoprotein
LDL: Low density lipoprotein
NCEP: National Cholesterol Education Program
NCEP-ATP III: National Cholesterol Education Program, Adult Treatment Panel III
TSH: Thyroid stimulating hormone
